# A case series of CHARGE syndrome: identification of key features for a neonatal diagnosis

**DOI:** 10.1186/s13052-020-0806-8

**Published:** 2020-04-23

**Authors:** Maria Francesca Bedeschi, Beatrice Letizia Crippa, Lorenzo Colombo, Martina Buscemi, Cesare Rossi, Roberta Villa, Silvana Gangi, Odoardo Picciolini, Claudia Cinnante, Viola Giulia Carlina Fergnani, Paola Francesca Ajmone, Elisa Scola, Fabio Triulzi, Fabio Mosca

**Affiliations:** 1 Fondazione IRCCS Ca’Granda Ospedale Maggiore Policlinico, Clinical Genetics Unit, Milan, Italy; 2 Fondazione IRCCS Ca’ Granda Ospedale Maggiore Policlinico, NICU, Milan, Italy; 3 Department of Clinical Sciences and Community Health, University of Milan, Milan, Italy; 4 Unit of Medical Genetics, Department of Medical and Surgical Sciences, Policlinico Sant’Orsola-Malpighi, University of Bologna, Bologna, Italy; 5 Fondazione IRCCS Ca’ Granda Ospedale Maggiore Policlinico, Pediatric Physical Medicine & Rehabilitation Unit, Milan, Italy; 6 Fondazione IRCCS Ca’ Granda Ospedale Maggiore Policlinico, Neuroradiology Unit, Milan, Italy; 7 Fondazione IRCCS Ca’ Granda Ospedale Maggiore Policlinico, Child and Adolescent Neuropsychiatric Service (UONPIA), Milan, Italy; 8 Department of Pathophysiology and Transplantation, Università Degli Studi Di Milano, Milan, Italy

**Keywords:** CHARGE syndrome, Early diagnosis, Ear malformations

## Abstract

**Background:**

An early diagnosis of CHARGE syndrome is challenging, especially for the primary care physicians who often take care of neonates with multiple congenital anomalies. Here we report eight cases of CHARGE syndrome whose diagnosis was made early in life with the intent to identify the most helpful features allowing a prompt clinical diagnosis.

**Methods:**

Medical records of patients with CHARGE syndrome whose diagnosis was made at the Fondazione IRCCS Ca′ Granda Ospedale Maggiore Policlinico in Milan, Italy were retrospectively reviewed.

**Results:**

Taken together, these patients reflect the considerable phenotypic variability of the syndrome; in one patient, the diagnosis was made immediately after birth because all the major criteria were met. In six patients, presenting with relatively nonspecific defects, a temporal bone computerized tomography scan was essential to achieve the correct diagnosis. In one patient, the diagnosis was made later than the others were. A careful examination revealed the presence of outer, middle, and inner ear anomalies: these elements, in the absence of any additional major criteria, represented for us an important diagnostic clue.

**Conclusions:**

This article suggests that an accurate evaluation of the ear should be made every time CHARGE syndrome is considered as a likely diagnosis even when the standard criteria are not fulfilled.

## Background

CHARGE syndrome (CS) (OMIM #214800) is an autosomal dominant condition with an occurrence of 1 in 10,000 births [1, 2]. The clinical features of CS were originally described in 1979 by Hall and Hittner [3, 4]. In 1981, Pagon et al. developed the CHARGE acronym (coloboma, heart defect, atresia choanae, retarded growth and development, genital hypoplasia, ear anomalies/deafness). Additional features of this syndrome include cleft lip and palate, hearing loss, tracheoesophageal fistula (TE), and cranial nerve dysfunction such as facial nerve palsy [5]. Some of the congenital abnormalities present in CS can lead to premature death [6].

At present, the clinical criteria elucidated by Blake and Verloes are used together with those of Hall and Hittner. The Blake criteria were slightly adjusted by a consortium and updated in 2009 and include four major and seven minor criteria with the major ones being abnormalities of the ear, coloboma, choanal atresia, cranial nerve dysfunction [5, 7]. Anomalies of the ear could potentially affect the external, internal and middle part with a frequency between 80 and 100% [8]. All four major, or three major and three minor, criteria must be present in order to diagnose CS. In 2005, Verloes proposed a revised set that included semicircular canal defects as a major criterion, anticipated broadening of the phenotypic spectrum, and reduced the number of features necessary for a diagnosis of CS [9]. Blake [5] and Verloes [9] criteria are summarized in Table 1.

**Table 1:** Blake and Verloes diagnostic criteria

	MAJOR CRITERA	MINOR CRITERIA	DIAGNOSIS
**Blake** [5]	Coloboma, microphthalmia Choanal atresia Ear abnormalities Cranial nerve dysfunction	Cardiovascular malformations Tracheoesophageal defects Genital hypoplasia/delayed pubertal development Cleft lip and/or palate Developmental delay Growth retardation Characteristic face	**Typical CHARGE** 4 major criteria 3 major + 3 minor criteria
**Verloes** [8]	Coloboma (iris or choroid) Choanal atresia Hypoplastic semicircular canals	Rhombencephalic dysfunction Hypothalamo-hypophyseal dysfunction Abnormal middle or external ear Malformation of mediastinal organs Mental retardation	**Typical CHARGE** 3 major criteria 2 major and 2 minor criteria **Partial/incomplete CHARGE** 2 major and 1 minor criteria **Atypical CHARGE** 2 major criteria 1 major and 3 minor criteria

CS was previously referred as an association until chromodomain helicase DNA binding protein 7 (*CHD7*), located on chromosome 8q12.1, was identified as the main gene responsible for the syndrome [10, 11]. Diagnosis now can be confirmed but not excluded by identifying a mutation of this gene found with a detection rate varying between 65 and 90% [8]. The condition is typically sporadic with few familial cases reported [7, 12]. It has a considerable phenotypic variability [2] with no single feature being consistently present and, for this reason, it represents a diagnostic challenge for the primary care physician. Here we report eight different cases of CS whose diagnosis was made early in life.

## Methods

We describe a series of eight patients with CS whose diagnosis was made in the Neonatal Intensive Care Unit and neonatal follow up service of our hospital from January 2012 to March 2018. Clinical data, imaging studies and laboratory test results were collected by consulting the infants’ computerized medical charts. All patients underwent a thorough clinical evaluation which included: echocardiography, abdominal ultrasonography, cerebral magnetic resonance imaging, cranial computed tomography (CT) (with the exception of patient 8), audiometry testing, fundoscopy, ear nose throat (ENT), neurological and genetic evaluation. Sequence analysis of the *CHD7* gene was performed in Policlinico Sant’Orsola-Malpighi in Bologna, Italy. The other genetic tests (i.e. karyotype and array-comparative genomic hybridization) were performed in our clinic. Informed consent was provided by both parents. The aim of this study was to identify, among all the clinical features, which were the most helpful in reaching the correct diagnosis and differentiating CS from other similar conditions.

## Results

In Table 2 we summarize the patients’ clinical features and molecular findings. Among major criteria, choanal atresia was detected only in patient 4. All patients presented with coloboma and hypoplastic or absent semicircular canals with the exception of patient 8 who presented instead with an abnormal right vestibular enlargement. Among minor criteria, rhombencephalic dysfunction, abnormal middle or external ear and psychomotor delay were reported in all patients. Malformation of mediastinal organs (i.e. heart and esophagus), to different degrees, were observed in seven patients.

**Table 2:** Clinical and genetic features

	Patient 1	Patient 2	Patient 3	Patient 4	Patient 5	Patient 6	Patient 7	Patient 8	Prevalence of clinical features	
									In our patients	In the literature^**8**^
**Presenting feature**	Esophageal atresia with fistula	Cleft lip and palate	Double outlet right ventricle, pulmonary valve stenosis, VSD, ASD	Blefarofimosis with microphthalmia and cyst, esophageal atresia with fistula	Axial hypotonia and hypertonia of extremities	Difficulty in sucking	Esophageal atresia with fistula	Difficulty in swallowing	NA	NA
**Ocular defects**	Bilateral chorioretinal coloboma	Bilateral chorioretinal coloboma	Bilateral chorioretinal coloboma	Bilateral coloboma, left blefarofimosis with microphthalmia and cyst	Bilateral chorioretinal coloboma	Left chorioretinal coloboma	Left chorioretinal coloboma	None	7/8	80–90%
**Choanal atresia**	No	No	No	Yes	No	No	No	No	1/8	50–60%
**Outer ear anomalies**	Squared ears absent lobules	Low set ears with antihelix anomalies	Dysplasia of ear pads	Low set squared ears	Dysplasia of ear pads, small lobules	Dysplasia of ear pads	Squared ears absent lobules	Low set ears with small lobules	8/8	80–100%
**Middle ear anomalies**	Ossicular malformation and right stenotic oval window	None	None	Right stapes and incus malformation and stenotic oval window	Bilateral stapes and incus malformation and atretic oval window	None	Right atretic oval window	Dysplasia of the stapes and of the oval window	5/8	80–100%
**Inner ear anomalies**	Bilateral hypoplasia of SCC and vestibulum and cochlear malformation (incomplete partition type II)	Right aplasia of superior and lateral SCC and hypoplasia of left superior and lateral SCC and bilateral posterior SCC. Left vestibular enlargement Bilateral cochlear malformation (incomplete partition type II) Bilateral stenotic Rosenthal’s canal	Bilateral absence of semicircular canals, bilateral cochlear malformation and vestibular dysplasia	Bilateral aplasia of SCC, Right cochlear malformation (incomplete partition type II) and vestibular dysplasia. Right stenotic Rosenthal’s canal	Bilateral absence of SCC, vestibular and cochlear malformation (incomplete partition type II). Bilateral aplasia of Rosenthal’s canal	Bilateral hypoplasia of lateral SCC and aplasia of posterior SCC	Bilateral aplasia of SCC	Bilateral aplasia of superior and posterior SCC, dysplasia of lateral SCC and vestibulum Abnormal right vestibular enlargement	8/8	80–100%
**Heart defects**	ASD	Pulmonary valve stenosis	Double outlet right ventricle, pulmonary valve stenosis, VSD, ASD	VSD	ASD and PDA	Pulmonary valve stenosis	None	None	6/8	75–85%
**Tracheoesophageal anomalies**	Esophageal atresia with fistula	None	None	Esophageal atresia with fistula	None	None	Esophageal atresia with fistula	None	3/8	15–20%
**Lip, palate, pharynx, larynx**	None	Cleft lip and palate	None	None	Velopharingeal insufficiency, hypotonia of vocal cord	None	None	Severe laryngomalacia and tracheomalacia	3/8	15–20%
**Genital anomalies**	None	None	None	Genital hypoplasia	None	Imperforate hymen	None	Micropenis, cryptorchidism,	4/8	50–60%
**Renal anomalies**	Multicystic left kidney	None	None	None	None	None	None	Horseshoe kidney	2/8	25–40%
**Brain anomalies**	Cerebellar vermis hypoplasia	Cerebellar vermis hypoplasia	None	Cerebellar vermis hypoplasia	Axial hypotonia and hypertonia of extremities	None	Cerebellar and pons hypoplasia	Hypotonia	6/8	NA
**Cranial nerve anomalies**	Hypoplasia of the olfactory bulbs, hypoplasia of optic nerves, bilateral neurosensorial hearing loss	Hypoplasia of the olfactory bulbs, bilateral neurosensorial hearing loss	Left laryngeal hemiplegia, peripheral paralysis of left facial nerve, deficit in swallowing, bilateral neurosensorial hearing loss	Agenesis of olfactory bulbs, hypoplasia of left optic nerve and of left part of optic chiasm, right aplasia of superior vestibular nerve	Agenesis of olfactory bulbs, hypoplasia of optic nerves, aplasia of vestibular nerves, bilateral neurosensorial hearing loss	Agenesis of olfactory bulbs, hypoplasia of optic nerves, bilateral, right cochlear nerve aplasia, neurosensorial hearing loss, deficit in swallowing and sucking	Hypoplasia of left olfactory bulbs, hypoplasia of optic nerves, deficit in swallowing	Depressor oris, deficit in swallowing, agenesis of olfactory right bulb, hypoplasia of optic nerves, left cochlear nerve aplasia, left neurosensorial hearing loss	8/8	70–90%
**Genetic tests performed before CHD7 analysis**	Karyotype in prenatal period	None	Karyotype array-CGH in prenatal period	None	None	None	None	FISH for 22q11.2, array-CGH, targeted NGS panel for Kallmann S.	NA	NA
**CHD7 mutation**	c.2867C > A; p.Ser956X unknown origin parents not available	c.8745–8746 insA fs2948X unknown origin parents not available	c.2429 C>G;p.Ser810Xfather wt; mother not available	c.5428C>T;p.Arg1810Xde novo origin	c.1465 C > T; p.Gln489X de novo origin	c. 5050 A > G; p.Gly1684Ser de novo origin	c.5884 G > A p.Gly1802Asp de novo origin	c.5405-17G > A; IVS25 de novo origin	NA	NA

*Array -CGH* array comparative genome hybridization, *ASD* atrial septal defect, *CHD7* chromodomain helicase DNA binding protein, *CNS* central nervous system, *NA* not applicable, *NGS* next generation sequencing, *PDA* patent ductus arteriosus, *SCC* semicircular canal, *VSD* ventricular septal defect

**Fig. 1:**
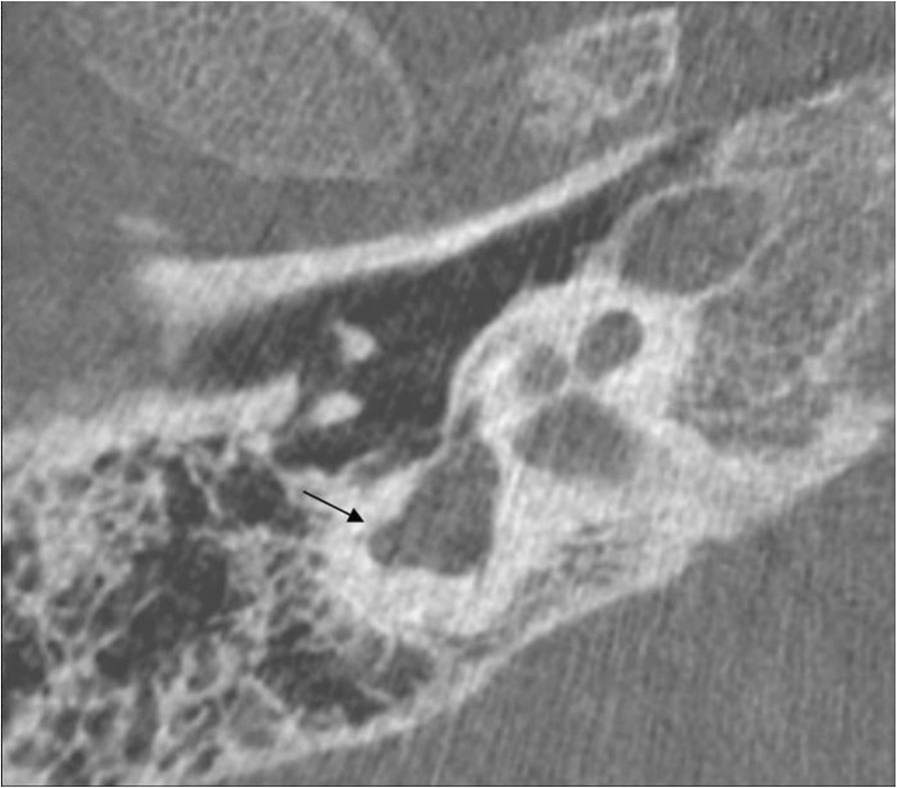
Patient 8’s axial CT image shows the dysplasia of lateral SCC (black arrow) which is only partially present

In patients 1–7, the clinical diagnosis of CS was made within the first month of life. On the other hand, the correct diagnosis in patient 8 was made at 18 months of life. Initially, 22q11.2 deletion syndrome was suspected because of the clinical presentation, in particular the marked difficulty in swallowing. Fluorescence in situ hybridization specific for 22q11.2 region along with array-comparative genomic hybridization were performed and both analysis resulted normal. Considering the findings of hypogonadotropic hypogonadism and agenesis of olfactory right bulb, a diagnosis of Kallmann Syndrome was proposed. Molecular analysis with next generation sequencing was also performed but no mutations were found for *KAL1*, *FGFR1*, *PROKR2*, *GnRHR*, *GnRH1*, *GnRH2*, *KISSR1*, *TAC3*, *TACR3*, or *HS6ST*. The correct diagnosis was finally achieved only by focusing on ear malformations: the patient presented with low set ears with small lobules along with abnormal right vestibular enlargement as seen by cerebral magnetic resonance imaging (MRI) and bilateral aplasia of superior and posterior semicircular canals as well as dysplasia of lateral semicircular canal and vestibulum, dysplasia of the stapes and of the oval window, depicted by CT (Fig. 1). CS was confirmed by molecular analysis of the *CHD7* gene which revealed a heterozygous mutation (c.5405-17G > A; IVS25). Patient 4 died at 6 months of age from cardiopulmonary arrest. The other patients are currently alive and all of them present with developmental delay and hearing impairment.

## Discussion

An early diagnosis of CS is important to enable the establishment of a multidisciplinary care team to manage the developmental concerns [12]. This syndrome has a considerable phenotypic variability [7] and many of its features including genital hypoplasia, cleft palate, and heart defect are shared with other syndromes such as 22q.11.2 deletion, Kallmann, Treacher Collins, and VACTERL (vertebral, anorectal, TE, renal and limb defects) [11–13]. Moreover, some clinical features may not be fully expressed early in life, some cannot be observed on physical examination, and mental retardation becomes evident only over time. For these reasons, a differential diagnosis can be challenging for the neonatologist who often takes care of newborns with multiple congenital anomalies. In our case series, rhombencephalic dysfunction and ear anomalies were reported in all patients. Multiple cranial nerve involvement produces many ENT concerns including olfactory, facial, glossopharyngeal and vagus nerve involvement. Moreover, choanal stenosis/atresia, cleft lip/palate and TE fistulas may also be present. For this reason, consultation by an ENT physician is essential.

For patient 4 all the major criteria were met and the diagnosis was made immediately after birth. Patients 1–3 and 5–7 presented with relatively nonspecific defects, except for bilateral coloboma and in all these patients, temporal bone CT scan was crucial to obtain the correct diagnosis. In fact, when Verloes proposed revised criteria, semicircular canal defects were included as a major one, as these defects were shown to be a very specific and consistent feature of CS [14, 15]. Patient 8 was the tricky one, and the diagnosis was made much later with respect to the others. He did not express any major criteria but had a significant feeding problem along with renal anomalies, hearing loss, hypogonadotropic hypogonadism, and agenesis of olfactory right bulb. For this reasons, 22.q11.2 deletion syndrome and Kallmann syndrome were initially suspected. In the diagnostic management of this case, focusing on ear anomalies was extremely helpful in pointing to the correct diagnosis. Although an abnormal right vestibular enlargement is not specific for CS, the presence of aplasia of semicircular canals together with the middle and outer ear anomalies was crucial in addressing the proper diagnosis.

**Fig. 2:**
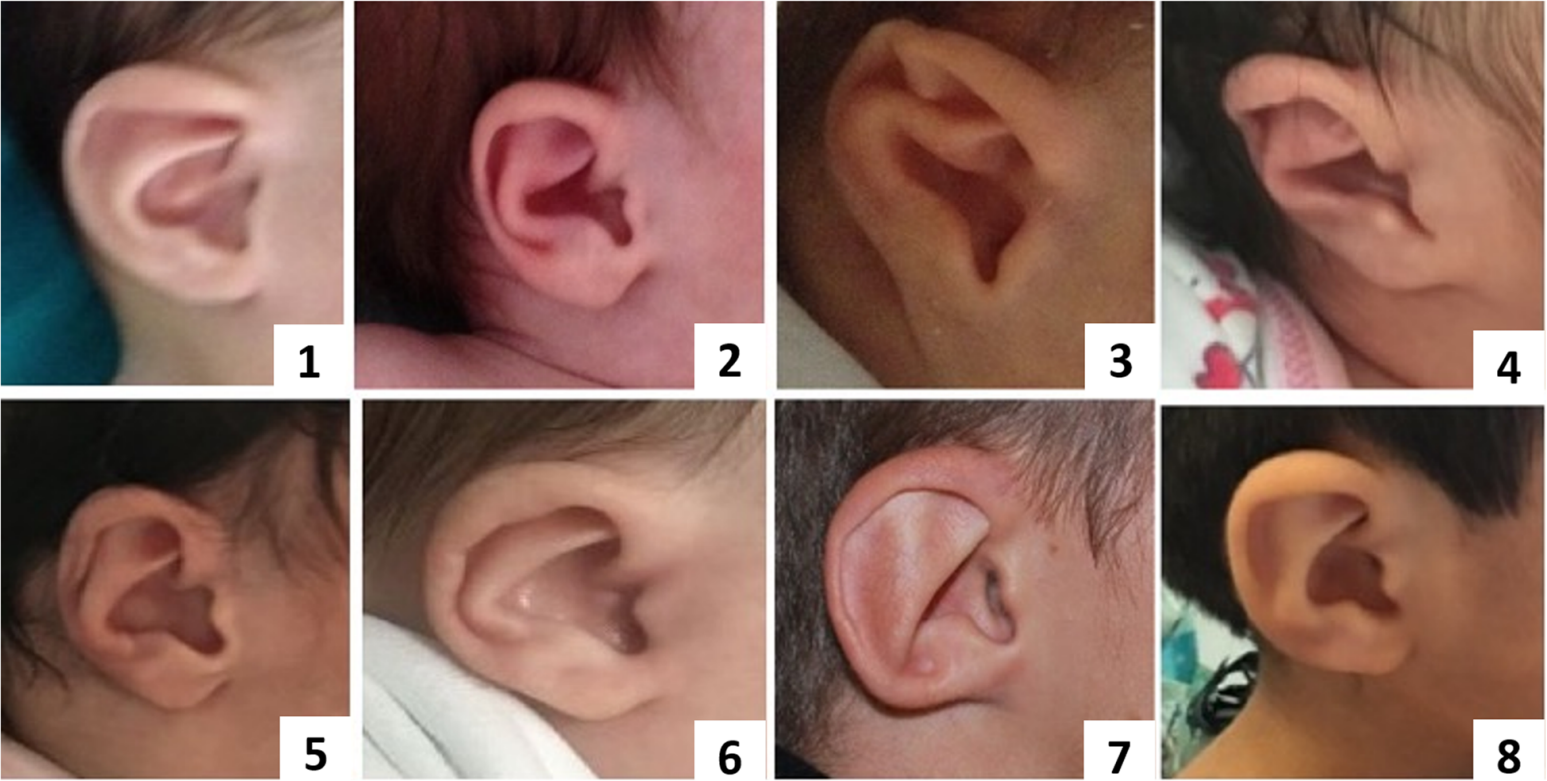
External typical aspect of ears in our patients

In CS, ear abnormalities are extremely frequent being found in > 90% of patients. Although semicircular canal anomalies are highly penetrant features in this syndrome, all the three segments of the ear can be affected and, in fact, ear anomalies are included both in major and in minor criteria [8, 16]. External malformations usually involve an abnormal shape and position of the pinnae, a cup shape wide helix, frequently small or absent lobules (Fig. 2). Middle ear involvement includes ossicular malformations, in particular the aplasia or dysplasia of the incus, of the stapes and oval and round windows, and chronic serus otitis which contributes to conductive hypoacusia [8, 16]. Inner ear abnormalities include cochlear and vestibular anomalies such as dysplasia of the vestibulum and varying degrees of cochlea hypoplasia and malformations, as well as aplasia or dysplasia of semicircular canals which is quite characteristic of CS [8, 16]. Patient 8 presented with low set ears with small lobules, a right mild conductive hearing loss, and an abnormal right vestibular enlargement detected by cerebral MRI as well as bilateral aplasia of semicircular canals. These elements reinforced the hypothesis of CS, which was confirmed on molecular analysis. It is interesting to note that the mutation detected in this case (c.5405-17G > A; IVS25) was previously reported as associated with a mild phenotype, especially in a familial case supporting genotype – phenotype correlation [17].

## Conclusion

An early pediatric clinical diagnosis of CS remains a complicated task [18]. Which anomaly or combination of anomalies carries the greatest diagnostic weight is not entirely clear. When CS is considered as a likely diagnosis but the criteria are not fulfilled, our experience suggests that a careful observation of the ear could be helpful. Moreover, a CT scan and a MRI of the temporal bone should be obtained to look for the suggestive middle and inner ear defects. Although it is known that with appropriate imaging, abnormalities of the semicircular canals are found in as many as 95% of CHARGE individuals [14, 19–23], this study underlines the importance of a prompt recognition of these signs in the neonatal age to make early diagnosis and timely care.

## Data Availability

No datasets were generated or analysed during the current study.

## Abbreviations

Array –CGH: array comparative genome hybridization
CS: CHARGE syndrome
TE: Tracheoesophageal fistula
CT: Cranial computed tomography
ENT: Ear nose throat
MRI: Magnetic resonance imaging
NGS: Next generation sequencing
ASD: Atrial septal defect
CHD7: Chromodomain helicase DNA binding protein
CNS: Central nervous system
PDA: Patent ductus arteriosus
SCC: Semicircular canal
VSD: Ventricular septal defect
